# Examining Teacher Self-Efficacy and Work Engagement Among Chinese School-Level EFL Teachers: The Mediating Roles of Teaching Anxiety and Enjoyment

**DOI:** 10.3390/bs16091640

**Published:** 2026-09-14

**Authors:** Zixin Xie, Yumei Fan

**Affiliations:** School of Foreign Languages, Central China Normal University, Wuhan 430079, China; zixinxie@mails.ccnu.edu.cn

**Keywords:** work engagement, self-efficacy, foreign language teaching anxiety, foreign language teaching enjoyment, English as a foreign language (EFL)

## Abstract

The affective turn in language education has prompted increasing scholarly attention to teachers’ emotional experiences. While teacher self-efficacy has been recognized as a predictor of work engagement, the mediating roles of specific teaching emotions remain underexplored. Grounded in the “EMPATHICS” framework, this study investigated how foreign language teaching anxiety (FLTA) and enjoyment (FLTE) mediate the relationship between self-efficacy and work engagement among school-level EFL teachers. Data from 288 EFL teachers in primary and secondary schools across China were analyzed using structural equation modeling with bias-corrected bootstrap mediation analysis (5000 resamples). Results showed that self-efficacy was positively associated with work engagement (*β* = 0.232, *p* = 0.023) and enjoyment (*β* = 0.640, *p* < 0.001), and negatively associated with anxiety (*β* = −0.507, *p* < 0.001). Both anxiety (*B* = 0.360, BC 95% CI [0.218, 0.573]) and enjoyment (*B* = 0.418, BC 95% CI [0.175, 0.779]) significantly mediated the relationship between self-efficacy and work engagement. These findings highlight the dual-affective mechanism linking self-efficacy to work engagement and underscore the value of fostering self-efficacy while addressing both teaching anxiety and enjoyment in school-based EFL contexts.

## 1. Introduction

Teaching is a demanding profession with heavy workloads, emotional challenges, and time pressures (Räsänen et al., 2020). In such a challenging context, teacher development and well-being are influenced by a range of internal and external factors (Cao, 2025; Geng & Han, 2025). Among these, teacher work engagement, defined as teachers’ investment and commitment to their work marked by vigor, dedication, and absorption (Schaufeli et al., 2006), is particularly critical. It reflects teachers’ deep cognitive and emotional connection to their work, directly fostering their well-being (Wang et al., 2022) and reducing turnover intentions (Høigaard et al., 2012; Räsänen et al., 2020). This importance extends to the context of language teaching, where language teachers face unique challenges related to language proficiency, pedagogical and emotional demands (Mișu et al., 2022; Y. Wu et al., 2026). Unlike teachers of other subjects, EFL teachers need to negotiate their dual roles as both language users and language instructors. Their professional self-appraisal, shaped by ongoing negotiation of language competence and pedagogical legitimacy, may influence their emotional experiences and commitment to teaching (Gong et al., 2025; Wang et al., 2025). Therefore, identifying the drivers of language teacher work engagement is essential for informing strategies to enhance instructional quality and support sustainable professional development (Cao, 2025).

Research on English as a foreign language (EFL) teachers has identified several antecedents of work engagement, such as self-efficacy (Zhang et al., 2023b), academic buoyancy (Zhi et al., 2024), grit (Fathi et al., 2023), professional agency (Cao, 2025), stress (L. Yang & Duan, 2024; Y. Wu et al., 2026), and loving pedagogy and emotion regulation (Wang et al., 2025; Derakhshan et al., 2023). While the positive correlation between teacher self-efficacy and work engagement is well-documented (H. Liu et al., 2024; Wang et al., 2025; Zhang et al., 2023b; Zhi et al., 2024), the mechanistic pathways linking these constructs, particularly through emotional pathways, remain insufficiently understood. To theorize this mechanism, this study draws on Positive Psychology (Seligman & Csikszentmihalyi, 2000) and, more specifically, on two dimensions of the “EMPATHICS” framework (Oxford, 2016): “Self-factors” (S) dimension corresponds to teacher self-efficacy, and the “Emotion” (E) dimension encompasses both positive and negative teaching emotions—namely, foreign language teaching enjoyment (FLTE) and anxiety (FLTA). These two dimensions thus offer a coherent theoretical lens for investigating how self-efficacy interacts with emotional experiences to shape work engagement.

While previous studies have examined the relationship between teacher self-efficacy and work engagement among Chinese EFL teachers (e.g., H. Liu et al., 2024; Wang et al., 2025), several research gaps remain. First, the relationship between negative teaching emotions (such as anxiety) and work engagement has been examined largely through qualitative research (e.g., Fraschini & Park, 2021) and lacks quantitative evidence. Second, while a few studies have jointly considered positive and negative teaching emotions, the existing literature offers limited insight into their simultaneous mediating roles between self-efficacy and work engagement. Third, few studies situate these cognitive and affective variables within an integrative framework such as “EMPATHICS”. Finally, most studies have focused on university-level EFL teachers (e.g., Geng & Han, 2025), with comparatively less attention given to school-level (K-12) EFL teachers in China whose classroom realities and professional challenges differ markedly from those in tertiary settings (S.-M. Wu, 2025).

To address these gaps, this study, drawing on two dimensions of the “EMPATHICS” framework (i.e., self-factors and emotions), aims to investigate the distinct emotional pathways linking self-efficacy to work engagement among a sample of Chinese school-level EFL teachers, with a specific focus on the roles of FLTE and FLTA. By doing so, the study seeks to provide a more integrated theoretical understanding and generate practical insights aimed at supporting the well-being and retention of this essential teacher population.

## 2. Literature Review

### 2.1. Theoretical Framework

This study is grounded in positive psychology (PP), which focuses on human strengths, well-being and flourishing (Seligman & Csikszentmihalyi, 2000), seeking to promote school teacher engagement by highlighting personal resources and emotions (Gregersen & Mercer, 2021, p. 64). Specifically, the study draws on the “EMPATHICS” framework (Oxford, 2016), an adapted version of PP’s PERMA framework, which provides a comprehensive perspective on the psychological aspects of language teachers and learners (Dewaele & MacIntyre, 2019; MacIntyre, 2021).

The EMPATHICS framework contains nine interconnected dimensions: emotion and empathy (E), meaning and motivation (M), perseverance (P), agency and autonomy (A), time (T), habits of mind (H), intelligence (I), character strengths (C), and self-factors (i.e., self-efficacy) (S). These dimensions are not discrete but dynamically interactive, meaning that change in one dimension may reverberate across others. For example, heightened emotional experiences (E) may reinforce or undermine a teacher’s sense of agency (A) and perseverance (P), while strong character strengths (C) may buffer against emotional exhaustion. Importantly, Self-factor (S), which encompasses self-efficacy, self-concept and self-esteem, is theorized to serve as a foundational resource that shapes how teachers appraise their professional environment and respond to challenges (Oxford, 2016). This systemic, dynamic perspective suggests that well-being emerges from the ongoing interplay among these dimensions rather than from any single component in isolation (Oxford, 2016).

In alignment with the research objectives and practical feasibility, this study focuses specifically on two dimensions, Emotion (E) and Self-factors (S), and their interplay. The self-factors (S) dimension shapes how teachers appraise their capabilities and navigate professional challenges. The Emotion (E) dimension captures teachers’ affective experiences, including positive emotions (e.g., enjoyment) and negative emotions (e.g., anxiety). Within the framework, Self-factors and Emotion are theorized to be mutually influential (Oxford, 2016). Teachers’ self-beliefs shape their emotional experiences, which in turn may feed back into their self-perceptions over time. Although this relationship may be reciprocal over time, the present study examines one theoretically specified pathway, in which self-efficacy (a core self-factor) is linked to work engagement through its association with emotional experiences (the Emotion dimension). While researchers acknowledge the integrative nature of the full EMPATHICS framework, the present study does not test the framework in its entirety but rather examines the relevance of its Self-factors and Emotion dimensions in the context of teacher work engagement.

### 2.2. Teacher Self-Efficacy and Work Engagement

Self-efficacy refers to an individual’s belief in their capability to execute a specific task (Bandura, 1977). An individual with high self-efficacy tends to experience more positive emotions and exhibit greater perseverance and determination when confronted with challenges (Bandura, 1977). In educational settings, Teacher Self-Efficacy (TSE) is regarded as the conviction of teachers in their capacity to perform teaching tasks and achieve desirable academic outcomes (Tschannen-Moran & Hoy, 2001).

Work engagement refers to “a positive, fulfilling, and work-related state of mind characterized by vigor, dedication, and absorption” (Schaufeli et al., 2006). Vigor stands for high energy and mental resilience at work; dedication is a sense of eagerness and inspiration while working; absorption is related to complete concentration on work (Hakanen et al., 2006). In teaching, Teacher Work Engagement (TWE) reflects teachers’ investment and commitment to their work, encompassing vigor, dedication, and absorption as well (Klassen et al., 2012).

Research shows that the relationship between teacher self-efficacy and work engagement has been established across both theoretical and empirical literature. As articulated in the “EMPATHICS” framework, higher self-efficacy is theorized to foster more positive and engaging experiences in language learning and teaching (Oxford, 2016). This theoretical proposition has been supported by empirical evidence, with multiple studies consistently identifying TSE as a significant positive predictor of TWE (e.g., H. Liu et al., 2024; Wang et al., 2025; Xu & Jia, 2022; Zhang et al., 2023a, 2023b; Zhi et al., 2024). For example, a study by Zhi et al. (2024) involving 242 EFL university teachers in China found that greater confidence in teaching correlated with higher levels of engagement in English instruction, a finding consistent with other studies (H. Liu et al., 2024; Wang et al., 2025; Xu & Jia, 2022). The consistent positive association observed across these studies can be understood through the self-efficacy theory (Bandura, 1977), which posits that teachers with stronger efficacy beliefs are more likely to perceive professional demands and obstacles as challenges to be mastered rather than as threats. This positive cognitive appraisal, in turn, enhances their satisfaction and sustained engagement in teaching (Wang et al., 2025). However, existing studies have been conducted primarily in tertiary education contexts, with comparatively less attention paid to this relationship among school-level EFL teachers, whose professional environments differ substantially from those in higher education. Consequently, a key objective of this study is to examine whether this association holds for a comparatively underexplored population of EFL teachers in Chinese primary and secondary schools.

### 2.3. Teacher Self-Efficacy and Foreign Language Teaching Emotions

Research has increasingly focused on the relationship between TSE and teachers’ emotional experiences, particularly within the demanding context of foreign language teaching (Zhang et al., 2023a, 2023b). With the advent of PP in educational research, researchers have adopted a holistic perspective on teacher emotions (Oxford, 2016), examining both positive and negative emotions in their research scopes (Seligman & Csikszentmihalyi, 2000). In second language teaching, the most frequently examined positive and negative emotions are Foreign Language Teaching Anxiety (FLTA) (Horwitz, 1996) and Foreign Language Teaching Enjoyment (FLTE) (Proietti Ergün & Dewaele, 2021). FLTA refers to the worries and fears that teachers experience during the process of foreign language teaching (Horwitz, 1996; M. Liu & Wu, 2021). For EFL teachers, such anxiety often stems from linguistic insecurity, self-doubt regarding their English proficiency, alongside other pedagogical concerns. FLTE is defined as broad positive emotions in this process, composed of teachers’ personal and social enjoyment and their feelings of student appreciation (Proietti Ergün & Dewaele, 2021). These two emotions represent the key foreign language teaching emotions explored in this study.

Theoretical support for the connection between self-efficacy and these emotions is provided by the “EMPATHICS” framework (Oxford, 2016), which posits that self-efficacy, as a key cognitive and personal factor (the “S” dimension), is intrinsically linked to emotional experiences (the “E” dimension). This aligns with Bandura’s (1977) theory of self-efficacy, which posits that efficacy beliefs shape emotional responses through cognitive appraisal processes. EFL teachers’ self-judgements about their language competence, shaped by societal expectations and professional identity concerns, may shape this appraisal process (Gong et al., 2025). Empirical studies have largely corroborated these theoretical associations (Bandura, 1997; Oxford, 2016), indicating a negative correlation between TSE and FLTA (Gong et al., 2025; M. Liu & Wu, 2021) and a positive correlation between TSE and FLTE (Zhang et al., 2023a). Collectively, these findings reveal that EFL teachers with strong efficacy tend to experience less anxiety and more enjoyment in their teaching practice (Zhang et al., 2023a). However, while the associations are established, the studies have ignored that positive and negative teaching emotions are predominantly examined in isolation. Most studies focus on either anxiety or enjoyment in relation to self-efficacy (Gong et al., 2025; M. Liu & Wu, 2021; Zhang et al., 2023a), failing to model them as parallel and potential interdependent pathways within a unified framework. It overlooks the complexity of both positive and negative emotions. Therefore, this study aims to address this gap by integrating both FLTA and FLTE for a more nuanced investigation of the emotional mechanisms linking TSE.

### 2.4. Foreign Language Teaching Emotions and Work Engagement

Beyond their links with self-efficacy, teaching emotions are theorized to play a direct and consequential role in shaping teacher work engagement (Y. G. Fan, 2022). The critical roles of positive and negative emotions in shaping one’s cognitive and behavioral repertoire (Fredrickson, 2001) have been substantiated by theoretical and empirical literature. The “EMPATHICS” framework underscores that effectively recognizing and managing emotions is vital for fostering positive interactions and engagement in educational settings (Oxford, 2016). This view aligns with the broaden-and-build theory (Fredrickson, 2001, 2003), which posits that positive emotions (i.e., FLTE) serve to expand an individual’s cognitive and behavioral resources, thereby facilitating engagement. Conversely, negative emotions (i.e., FLTA) have the opposite effect of narrowing these resources, potentially undermining engagement (Fredrickson, 2001, 2003).

Empirical research in language teaching provides clear support for the role of positive emotions. Studies have revealed a positive association between FLTE and TWE (Fathi et al., 2023; J. Fan et al., 2024; Xiao et al., 2022). For instance, Fathi et al. (2023) surveyed 476 EFL teachers at different institutions and schools in Iran and found that FLTE positively and directly predicted TWE. These findings are consistent with the theoretical premise that enjoyable teaching experiences fuel teachers’ energy, dedication and absorption in their work (S. Yang et al., 2023).

In contrast, the empirical research regarding negative emotions is underdeveloped. While the “EMPATHICS” framework and the broaden-and-build theory logically suggest a detrimental effect of anxiety on engagement, quantitative studies directly associating FLTA with TWE remain scarce. Limited empirical literature remains on qualitative insights, such as Fraschini and Park (2021), which hints at the negative impact of anxiety among university language teachers. Also, there is insufficient account for the school-level EFL teaching context. Therefore, this study aims to address these gaps by modelling FLTA and FLTE as parallel mediators in the relationship between TSE and TWE.

## 3. The Conceptual Model

The preceding review points to four gaps in the literature: First, there is still limited quantitative research on how FLTA relates to TWE. Second, while self-efficacy and work engagement have been studied, positive and negative teaching emotions (i.e., FLTA and FLTE) are rarely examined together as parallel pathways in the relationships between TSE and TWE. Third, these cognitive and affective variables (i.e., TSE, FLTA and FLTE) have rarely been examined together within a framework that specifies their interrelationships, such as the Self-factors and Emotion dimensions of the EMPATHICS model. Finally, existing studies on Chinese EFL teachers have drawn predominantly on samples from tertiary settings, leaving school-level EFL teachers comparatively underexplored.

**Figure 1:**
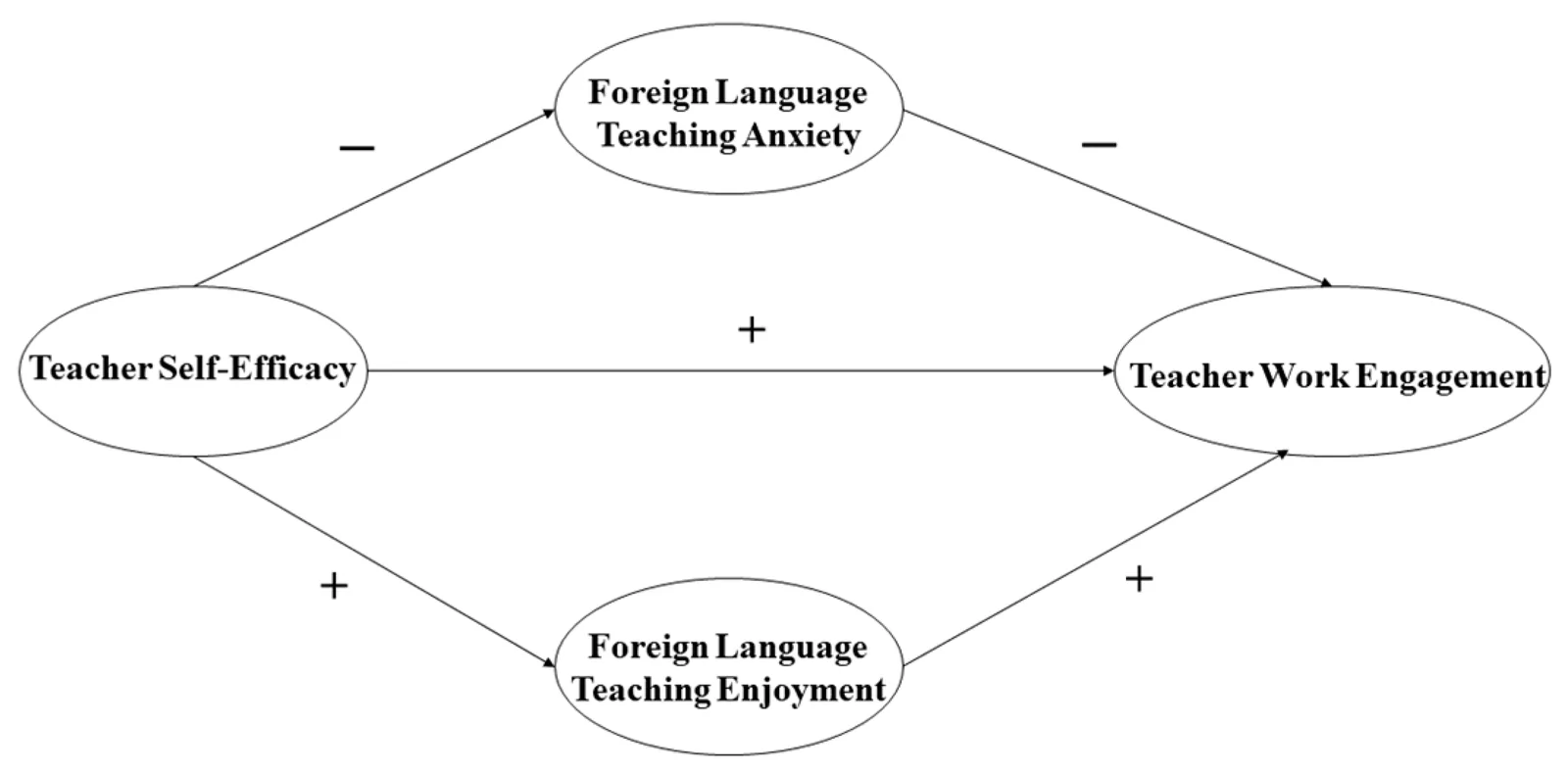
The hypothesized model of teacher work engagement.

To address these gaps cohesively, this study proposes a parallel mediation model (see Figure 1) to test the intrinsic associations between TSE, FLTA, FLTE and TWE. In this model, FLTA and FLTE are specified as parallel mediators. A key theoretical decision in this specification is whether the two emotions should be treated as correlated but distinct constructs, or as opposite ends of a single bipolar continuum. FLTA and FLTE are conceptualized as distinct emotional experiences that may co-occur, rather than as a single bipolar affect construct, for two reasons. Theoretically, FLTA and FLTE represent qualitatively different emotional responses to teaching and teachers may simultaneously experience both emotions in their professional practice (Dewaele & MacIntyre, 2019). Empirically, a parallel mediation allows for the estimation of their unique mediating effects while accounting for their shared variance. A sequential mediation model, by contrast, would impose a directional ordering between FLTA and FLTE that is not clearly specified by the EMPATHICS framework (Oxford, 2016). Based on the proposed model, six hypotheses are as follows:

**Hypothesis** **1.** *Teacher self-efficacy positively influences teacher work engagement.*

**Hypothesis** **2.** *Teacher self-efficacy negatively influences foreign language teaching anxiety.*

**Hypothesis** **3.** *Teacher self-efficacy positively influences foreign language teaching enjoyment.*

**Hypothesis** **4.** *Foreign language teaching enjoyment positively influences teacher work engagement.*

**Hypothesis** **5.** *Foreign language teaching anxiety negatively influences teacher work engagement.*

**Hypothesis** **6.** *Foreign language teaching anxiety and enjoyment play parallel mediating roles in the relationship between teacher self-efficacy and work engagement.*

## 4. Methodology

This study employed a quantitative, cross-sectional, nonexperimental survey design. The primary objective was to test a parallel mediation model examining the relationships among teacher self-efficacy, teaching emotions (anxiety and enjoyment), and work engagement. The study is hypothesis-driven and therefore does not include separate research questions.

### 4.1. Participants

This study employed convenience and snowball sampling methods (Dörnyei, 2007) to recruit participants, a strategy adopted because of the practical challenges involved in accessing a large and geographically dispersed population of in-service school EFL teachers across China, for which no comprehensive national sampling frame was readily available. Recruitment targeted in-service EFL teachers from primary and secondary schools in China. Invitations were initially distributed via the researchers’ professional and academic networks. Participants were then encouraged to forward the survey link to other eligible colleagues, facilitating the snowball process. This approach enabled efficient data collection across multiple provinces within a limited timeframe. It is, however, inherently selective, which we acknowledge as a limitation.

A total of 305 EFL teachers from primary and secondary schools in various provinces in China voluntarily participated. After initial scrutiny, which excluded 17 respondents as identical responses across all items, completion time less than 180 s, or a failure to complete over 95% of the survey items, 288 valid responses were obtained (66 males, 222 females). Of those, 199 teachers were from urban schools, while 89 teachers came from rural schools. Regarding their school types, 101 teachers were from primary schools, 107 teachers came from junior middle schools, and 80 teachers were from senior high schools. Their teaching experience ranged from 1 to 3 years to more than 30 years, with the vast majority of teachers (115 teachers) having 1–3 years. In addition, all the participants majored in English at university, including 170 teachers with a B.A. degree, 95 teachers with an M.A. or M.E. degree, and 23 teachers with a Ph.D. or Ed.D. degree. The final sample size of 288 is methodologically sufficient, meeting established guidelines for structural equation modeling regarding both model complexity and statistical power (Kline, 2016; Schreiber et al., 2006). Detailed demographic information of the participants is presented in Table 1.

### 4.2. Research Instruments

A composite questionnaire was used to collect quantitative data, which consisted of five sections: demographic information, teacher self-efficacy scale, foreign language teaching anxiety scale, foreign language enjoyment scale, and teacher work engagement scale. All scales were adapted through a careful translation and validation procedure. First, the original instruments were independently forward-translated into Chinese by the first author with consideration of the context of English language teaching in China. Second, the Chinese versions were back-translated into English by the second author, a professor of linguistics, who was blind to the original versions, to ensure semantic and conceptual equivalence. Third, the two translators compared the original and back-translated versions and resolved any discrepancies through discussion. Fourth, the resulting drafts were reviewed by a bilingual expert in applied linguistics to verify cultural and contextual appropriateness. Finally, the preliminary Chinese versions were pilot-tested with 10 in-service secondary school EFL teachers, whose feedback on clarity and relevance was incorporated into the final survey before formal distribution. The following sections provide detailed information on the four measurement scales:

Teacher Self-efficacy Scale: In this study, TSE was assessed using the 12-item short form of the Teachers’ Sense of Efficacy Scale (TSES) (Tschannen-Moran & Hoy, 2001), which was developed alongside the original 24-item long form in the same validation study. The short version scale contained 12 items (e.g., *“I can make students value English learning”*) covering three aspects, i.e., using effective teaching strategies, engaging students, and classroom management. Each item was assessed on a 5-point Likert scale, ranging from 1, “nothing”, to 5, “a great deal”. The validity of the scale was confirmed by scholars in different countries (Tschannen-Moran & Hoy, 2001). In this study, the internal consistency of the scale was 0.944.

Foreign Language Teaching Anxiety Scale: The study adapted the Foreign Language Teaching Anxiety Scale (FLTAS) (Kim & Kim, 2004) to measure FLTA in the EFL Teaching context in China. The scale reported a high level of reliability and validity in previous studies, containing four dimensions: limited knowledge, limited language skills, teaching situations, and fear of negative evaluation (Kim & Kim, 2004). After the pilot phase, the questionnaire was revised to include 12 items based on the feedback from the participants within the Chinese context, including items such as *“I feel uneasy if students get bored in my class”.* Each item was assessed on a five-point Likert scale varying from 1, “strongly disagree” to 5, “strongly agree”. In this study, the internal consistency of the scale was 0.931.

Foreign Language Teaching Enjoyment Scale: The Foreign Language Teaching Enjoyment Scale (FLTES) (Proietti Ergün & Dewaele, 2021) was used to measure FLTE. This scale includes 9 items (e.g., *“In a positive classroom atmosphere, I always laugh with my students.”*) assessing three underlying components: personal Enjoyment (PE), Student Appreciation (SA), and Social Enjoyment (SE). Each item was rated using a 5-point Likert scale from 1, “strongly disagree”, to 5, “strongly agree”. Previous studies (e.g., Proietti Ergün & Dewaele, 2021; Zhang et al., 2023b) reported its high internal consistency. In this study, the scale achieved an internal consistency of 0.910.

Teacher Work Engagement Scale: TWE was measured using the short-form Utrecht Work Engagement Scale (UWES-9; Schaufeli et al., 2006). The 9-item scale assesses the dimensions of Vigor, Dedication, and Absorption and includes items such as *“I am fully engaged in my work at all times.”* All items were rated on a 7-point Likert scale ranging from 1 (never) to 7 (always). Considerable previous research (e.g., Klassen et al., 2012; Wang et al., 2022) has examined the scale with teachers in educational settings and confirmed the reliability and validity of the scale. In this study, the internal consistency of the scale was 0.932.

### 4.3. Data Collection

Data collection was conducted over about two months, starting in December 2023, allowing participants ample time to complete the survey at their convenience (Zhang et al., 2023b). The researchers designed an electronic survey using the WenJuanXing (web version) and then distributed the link through two major social network platforms (i.e., WeChat 8.0.77 and QQ v 9.3.55.40650) to EFL teachers from different backgrounds in China. In addition, some teacher educators voluntarily contributed to the data collection efforts by sharing the survey link with their professional networks of EFL school teachers. Participants were also encouraged to share the link with eligible colleagues to expand the sample size. Before participating in the survey, participants provided consent, were informed of the research purpose, and were assured that their data would be kept confidential.

### 4.4. Data Analysis

This study utilized IBM SPSS Statistics 27 and AMOS 26 for statistical analysis. Data quality checks were meticulously performed, including a thorough examination for missing data, outliers, and normality (Tabachnick & Fidell, 2013). Additionally, Harman’s single-factor test was conducted as an initial diagnostic for common method bias (CMB) (Podsakoff et al., 2003). Descriptive and correlation statistical analyses were then conducted to provide an overview of the data.

To evaluate the psychometric properties of the research instruments, researchers utilized Confirmatory Factor Analysis (CFA) on four separate occasions. The goodness-of-fit of the measurement models was assessed using the following indices (Kline, 2016): Chi-square divided by degrees of freedom (χ^2^/df), which evaluates overall model fit with lower values indicating better fit; Comparative Fit Index (CFI) and Tucker–Lewis Index (TLI), which compare the proposed model to a baseline model, with values closer to 1.00 representing superior fit; and Root Mean Square Error of Approximation (RMSEA), which assesses approximate fit, with values ≤ 0.08 suggesting acceptable error. The adopted thresholds for a well-fitting model were χ^2^/df < 3, CFI and TLI ≥ 0.90, and RMSEA ≤ 0.08 (Kline, 2016). Furthermore, the convergent validity and reliability of the constructs were examined using average variance extracted (AVE) and composite reliability (CR). To confirm the distinctiveness of the four latent constructs, the proposed four-factor model was compared against plausible alternative factor structures (one-factor, two-factor, and three-factor models).

Based on these foundations, Structural Equation Modeling (SEM) was then applied to test the hypothesized parallel mediation model. SEM was considered appropriate for this study as it allows for the simultaneous estimation of multiple direct and indirect pathways among latent constructs (Kline, 2016). The structural model included all hypothesized direct paths (TSE → FLTA, TSE → FLTE, TSE → TWE, FLTA → TWE, FLTE → TWE), and the residuals of FLTA and FLTE were allowed to covary, given their conceptual and empirical relatedness. This standardized residual association was statistically significant (r = −0.249, BC 95% CI [−0.418, −0.070], *p* = 0.005). The structural model was saturated relative to the measurement model, as all possible direct paths among the constructs were estimated.

To examine the significance of indirect effects, bootstrapping with 5000 bootstrap samples was employed (Hayes, 2013). Bias-corrected 95% confidence intervals were used to determine the significance of specific indirect effects, with intervals not containing zero indicating statistically significant mediation. In addition, a formal bootstrap contrast test was conducted to compare the two specific indirect effects (via FLTA vs. via FLTE) by defining a user-defined estimand representing their difference. Both standardized and unstandardized coefficients are reported, with standardized coefficients used to compare the relative magnitude of effects and unstandardized coefficients used to construct the bootstrap confidence intervals (Kline, 2016).

## 5. Results

Before proceeding with the analysis, the data underwent a screening process for missing data, outliers, and normality. Missing data were addressed using the expectation-maximization (EM) algorithm (Kline, 2016). Univariate and multivariate outliers were identified using standard scores and Mahalanobis D^2^, respectively (Tabachnick & Fidell, 2013). Normality was evaluated by examining skewness and kurtosis indices, with non-normal values falling beyond the ± 2.0 range (Kunnan, 1998). This initial scrutiny yielded 288 valid cases suitable for further analysis. Harman’s single-factor test was then conducted as an initial diagnostic for common method bias (CMB). The results showed that the first unrotated factor accounted for 40.39% of the variance, below the 50% threshold (Fuller et al., 2016; Podsakoff et al., 2003).

Table 2 demonstrates the descriptive statistics, correlations, and discriminant validity for the research variables. The descriptive analysis results revealed that participants experienced a moderate-to-high level of TSE (M = 3.59, 71.8% of the maximum score of 5) and FLTE (M = 3.69, 73.8% of 5), a relatively low level of FLTA (M = 2.51, 50.2% of 5), and a high level of TWE (M = 4.51, 64.4% of the maximum score of 7). The correlation analysis showed that TSE was significantly and positively correlated with both FLTE (*r* = 0.640, *p* < 0.001) and TWE (*r* = 0.639, *p* < 0.001). Additionally, a significant and negative correlation was found between TSE and FLTA (*r* = −0.507, *p* < 0.01). FLTA also displayed a significant negative correlation with TWE (*r* = −0.657, *p* < 0.001), while FLTE was significantly and positively correlated with TWE (*r* = 0.673, *p* < 0.001). The diagonal values in Table 2 (in bold) represent the square roots of the AVE for each construct, which are used to assess discriminant validity (Kline, 2016). All √AVE values exceeded the corresponding inter-construct correlations, supporting the distinctiveness of the four latent constructs.

Subsequently, CFA was performed separately for each instrument to evaluate their psychometric properties. As shown in Table 3, each of the four measurement models demonstrated acceptable to good fit. TSE (χ^2^/df = 2.615, CFI = 0.963, RMSEA = 0.075), FLTA (χ^2^/df = 2.778, CFI = 0.951, RMSEA = 0.079), FLTE (χ^2^/df = 2.223, CFI = 0.975, RMSEA = 0.065), and TWE (χ^2^/df = 2.155, CFI = 0.981, RMSEA = 0.063). All AVE values exceeded 0.50, and all CR values exceeded 0.70, supporting convergent validity and internal consistency reliability (Kline, 2016).

A four-factor measurement model comprising all 42 items was then tested. Standardized factor loadings for all items ranged from 0.618 to 0.847 and were all significant at *p* < 0.001. (The full item-level loadings are presented in Appendix A). The model demonstrated acceptable fit: χ^2^/df = 1.410 < 3, CFI = 0.957 > 0.90, TLI = 0.955 > 0.90, RMSEA = 0.038 < 0.08 (Kline, 2016). All residual variances were positive and statistically significant (*p* < 0.001), indicating no Heywood cases. To confirm the superiority of the four-factor structure, the model was compared against three plausible alternatives: a one-factor model (all items loading onto a single factor), a two-factor model (TSE + TWE vs. FLTA + FLTE), and a three-factor model (FLTA + FLTE merged as a single emotion factor, with TSE and TWE separate). As shown in Table 4, the four-factor model outperformed all alternatives, supporting the distinctiveness of the four constructs. In addition, discriminant validity was further supported by the Fornell-Larcker criterion (√AVE > inter-construct correlations, see Table 2), confirming the empirical distinctiveness of the four constructs.

**Figure 2:**
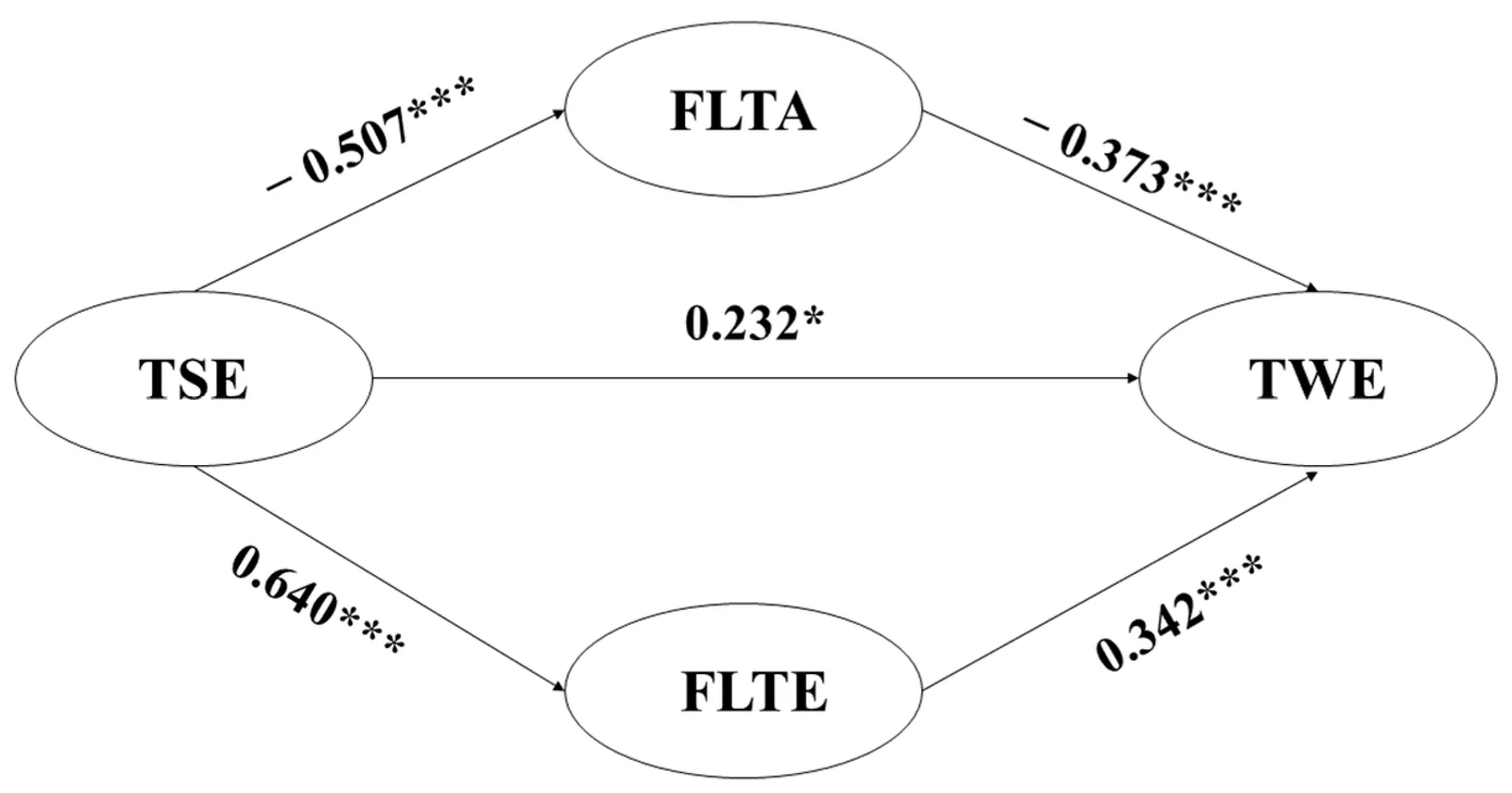
The final model of teacher work engagement Note. *** *p* < 0.001; * *p* < 0.05 (bootstrap-corrected); TSE = teacher self-efficacy. FLTA = foreign language teaching anxiety; FLTE = foreign language teaching enjoyment; TWE = teacher work engagement. The standardized residual covariance between FLTA and FLTE was freely estimated (r = −0.249, *p* = 0.005).

SEM was carried out to assess the hypothesized parallel mediation model. The model demonstrated an acceptable fit to the data: χ^2^/df = 1.410 < 3; CFI = 0.957 > 0.90, TLI = 0.955 > 0.90, RMSEA = 0.038 < 0.08 (Kline, 2016). Beyond overall fit, the model also explained substantial variance in the endogenous variables, accounting for 25.7% of the variance in FLTA, 41.0% in FLTE, and 62.3% in TWE. Figure 2 presents the final model of teacher work engagement.

The analysis revealed significant direct effects of TSE on FLTA, FLTE and TWE and significant direct effects of FLTA and FLTE on TWE (H1-H5 supported). Table 5 summarizes the direct effects of the SEM analysis, including bias-corrected bootstrap 95% confidence intervals. Participants with higher TSE scores reported lower FLTA (*β* = −0.507, BC 95% CI [−0.622, −0.375], *p* < 0.001) (H2 supported), higher FLTE (*β* = 0.640, BC 95% CI [0.498, 0.763], *p* < 0.001) (H3 supported), and greater TWE (*β* = 0.232, BC 95% CI [0.029, 0.452], *p* = 0.023) (H1 supported). These findings suggest that TSE is negatively associated with FLTA and positively associated with FLTE and TWE. Specifically, the large standardized path coefficient (*β* = −0.507) for the relationship between TSE and FLTA indicates a strong negative association, while the positive association between TSE and FLTE (*β* = 0.640) and TWE (*β* = 0.232) suggests meaningful positive associations with emotional engagement and work involvement. Similarly, FLTA (*β* = −0.373, BC 95% CI [−0.515, −0.228], *p* < 0.001) (H5 supported) and FLTE (*β* = 0.342, BC 95% CI [0.142, 0.561], *p* = 0.001) (H4 supported) both had significant direct effects on TWE, suggesting their independent associations with this outcome.

To enhance confidence in the indirect effects of the mediation analysis, a bootstrapping technique with 5000 iterations was implemented (Hayes, 2013), yielding more reliable estimates of standard errors and confidence intervals (Hayes, 2013). A significant indirect effect is indicated when its 95% CI does not span zero. Table 6 presents the results of the mediation analysis. The results showed significant indirect effects of TSE on TWE, mediated by FLTA and FLTE (H6 supported). TSE had a significant indirect effect on TWE through FLTA (*β* = 0.189, *B* = 0.360, BC 95% CI [0.218, 0.573], *p* < 0.001) and through FLTE (*β* = 0.219, *B* = 0.418, BC 95% CI [0.175, 0.779], *p* = 0.001). The total indirect effect was also significant (*β* = 0.408, *B* = 0.779, BC 95% CI [0.489, 1.170], *p* < 0.001). Given that the direct effect of TSE on TWE remained significant (*β* = 0.232, *p* = 0.023) alongside the significant indirect effects, the results indicate partial mediation rather than complete mediation. That is, TSE was associated with TWE both directly and indirectly via the two emotional mediators. A bootstrap contrast test revealed that the two indirect effects did not differ significantly, as the bias-corrected 95% CI for their difference crossed zero ([−0.266, 0.457], *p* = 0.791). This suggests that FLTA and FLTE play comparable mediating roles in the relationship between TSE and TWE.

Furthermore, the total effect of TSE on TWE (*β* = 0.639, *B* = 1.221, BC 95% CI [0.957, 1.535], *p* < 0.001) was significant, integrating both direct and indirect pathways. This suggests that TSE is associated with TWE both directly and through its associations with teaching emotions. These findings highlight the potential relevance of TSE and teaching emotions to teachers’ work engagement and suggest that these factors may warrant attention in teacher training and professional development.

## 6. Discussion

Based on two dimensions of the “EMPATHICS” framework (i.e., Self-factors (S) and Emotion (E) (Oxford, 2016), this study examined how TSE is associated with TWE through FLTA and FLTE as mediators among a sample of Chinese EFL teachers from primary and secondary schools. These findings revealed the interplay between the “Self-factors” (S) and “Emotion” dimensions in the EMPATHICS framework, offering insights into the understanding of how cognitive and affective processes jointly shape teacher engagement (MacIntyre, 2021). It should be noted, however, that this study does not test the framework in its entirety. Rather, it offers partial empirical support for the relevance of the Self-factors and Emotion dimensions within this framework.

First, the findings support hypothesis 1, demonstrating a direct positive association between TSE and TWE. The result underscores that participants with higher self-efficacy are more likely to appraise challenges as manageable, thereby conserving emotional and cognitive resources that can be channeled into sustained vigor, dedication and absorption in their work (Bandura, 1997; Oxford, 2016). This study aligns with recent multinational evidence reported by Wang et al. (2025), who identified self-efficacy as a significant predictor of work engagement among EFL teachers across China, Iran, and Turkey, supporting the generalizability of this relationship across different educational contexts. The results are also consistent with prior studies that have established the predictive role of self-efficacy in teacher work engagement (e.g., H. Liu et al., 2024; Xu & Jia, 2022; Zhang et al., 2023a, 2023b; Zhi et al., 2024), while extending this line of research to the school-level language teaching context. Notably, the total association between TSE and TWE in this study (*β* = 0.639) is numerically stronger than those reported in studies of university-level EFL teachers (e.g., *β* = 0.480, Wang et al., 2025; *β* = 0.320, Zhi et al., 2024). This difference may reflect the distinct professional context of school-level EFL teachers, who face sustained classroom and curricular pressures that may make self-efficacy a more immediate psychological resource. Despite methodological differences, this comparison suggests that the strength of the efficacy–engagement link may vary across educational stages, underscoring the need for context-sensitive teacher development support.

Second, the findings support hypotheses 2 and 3, which proposed that TSE is negatively associated with FLTA and positively associated with FLTE. The strong associations of TSE with FLTA (*β* = −0.507) and FLTE (*β* = 0.640) underscore the affective dimension of teacher self-efficacy (Bandura, 1997). The results align with previous studies (Gong et al., 2025; M. Liu & Wu, 2021; Zhang et al., 2023b) in that they illustrate how efficacy beliefs may serve as an emotional filter in daily teaching. For example, teachers with stronger self-efficacy tend to appraise teaching situations as less threatening and more rewarding, thereby experiencing lower anxiety and greater enjoyment. This aligns with recent findings by Gong et al. (2025) on Chinese university EFL teachers, who reported that low self-efficacy was associated with greater teaching anxiety, particularly with respect to self-perceived teaching competence and professional identity. It also echoes Zhang et al.’s (2023b) findings that self-efficacy is positively associated with FLTE among university foreign language teachers. Together, this dual affective pathway clarifies the mechanism implied in the “EMPATHICS” framework that self-factors do not reside in an emotional vacuum but are actively associated with the emotional experiences of foreign language teaching (Oxford, 2016).

Furthermore, the study reinforces the notion that FLTA and FLTE are directly associated with TWE, thus supporting hypotheses 4 and 5. This is consistent with the broaden-and-build theory (Fredrickson, 2001, 2003) and the Emotion dimension of the EMPATHICS framework (Oxford, 2016). The negative association between FLTA and TWE provides quantitative evidence, aligning in part with previous findings that highlighted the detrimental association of anxiety with teachers’ performance and commitment (Høigaard et al., 2012; Y. G. Fan, 2022; Fraschini & Park, 2021; L. Yang & Duan, 2024). This can be interpreted through the broaden-and-build theory, which suggests that anxiety may narrow teachers’ cognitive-behavioral repertoire, reducing the resources necessary for engaged teaching (Fredrickson, 2001, 2003). Conversely, FLTE demonstrated a robust positive association with TWE, corroborating previous studies on the role of enjoyment in supporting teacher engagement (J. Fan et al., 2024; Fathi et al., 2023; Xiao et al., 2022; Zhang et al., 2023b). In line with the broaden-and-build theory, enjoyment may expand teachers’ thought-action repertoires, build enduring personal resources, and enhance their capacity for dedicated and absorbed teaching (Fredrickson, 2001, 2003). This affective upward spiral aligns with the principles of positive psychology, suggesting that positive emotions are associated with professional motivation and commitment (MacIntyre, 2021; Oxford, 2016). These findings underscore the importance of giving equal attention to the roles of both positive and negative emotions in teacher development (Dewaele & MacIntyre, 2019).

Finally, the study demonstrates significant indirect effects of TSE on TWE, mediated by FLTA and FLTE, thereby supporting hypothesis 6. Specifically, the results revealed two novel pathways. On the one hand, lower TSE may be associated with higher FLTA, which may in turn be associated with reduced TWE among participants. As previously discussed, TSE is directly and negatively associated with FLTA but positively associated with TWE. Notably, FLTA appears to act as a negative emotional pathway in the association between TSE and TWE. This suggests that when participants experience self-doubt and diminished professional confidence, their teaching anxiety tends to be higher, a pattern consistent with Gong et al. (2025). Such heightened anxiety may, in turn, dampen their teaching enthusiasm and undermine work engagement, corroborating previous findings (e.g., Xu & Jia, 2022; L. Yang & Duan, 2024). Therefore, it may be beneficial to provide professional development programs to boost teachers’ confidence in teaching, with the aim of reducing anxiety and supporting greater dedication and involvement in teaching.

Conversely, the second pathway reveals how TSE is associated with a more positive and fulfilling work experience for EFL teachers. Previous studies have established that TSE predicts teaching enjoyment (Zhang et al., 2023a) and that positive emotions, including enjoyment, mediate the relationship between self-efficacy and work engagement (S. Yang et al., 2023). Consistent with this line of research, the present findings confirm that TSE is positively associated with FLTE, which in turn links to higher TWE. In this pathway, self-efficacy may serve as fertile ground for stimulating positive emotions (i.e., FLTE) in teachers’ daily professional activities (Fredrickson, 2003), which may support teachers’ commitment to their work (J. Fan et al., 2024; S. Yang et al., 2023).

A formal bootstrap contrast test revealed that the two indirect effects did not differ significantly, suggesting that anxiety reduction and enjoyment enhancement contribute to the TSE–TWE association with comparable strength. This finding aligns with the interconnected nature of the EMPATHICS framework (Oxford, 2016), which conceptualizes well-being as emerging from the dynamic interaction among its nine dimensions. The results empirically illustrate how the Self-factors (S) and Emotion (E) dimensions may operate in tandem, i.e., through lower negative affect and greater positive affect, in relation to teacher work engagement. This also resonates with the broaden-and-build theory (Fredrickson, 2001), which acknowledges that while positive emotions broaden cognitive and behavioral repertoires, the reduction in negative emotions may similarly free up resources by removing psychological barriers. Importantly, the comparable strength of the two pathways does not diminish the relevance of either emotion. Rather, it underscores the need for interventions that simultaneously address both anxiety reduction and enjoyment enhancement.

## 7. Conclusions and Implications

The study, grounded in the “EMPATHICS” framework (Oxford, 2016), examined a parallel mediation model to investigate the affective mechanisms linking teacher self-efficacy to work engagement among a sample of EFL teachers from primary and secondary schools in China. The results indicate that TSE is significantly associated with TWE both directly and indirectly through two emotional pathways, i.e., higher FLTE and lower FLTA. The two indirect effects were comparable, indicating that the two emotional pathways were similar in magnitude. This research provides empirical evidence for the relevance of the Self-factors (S) and Emotion (E) dimensions within the “EMPATHICS” framework by examining their interplay in relation to teacher work engagement. It extends previous studies by showing that self-efficacy is associated with both positive and negative teaching emotions as well as work engagement, and by demonstrating that both anxiety reduction and enjoyment enhancement serve as comparable mediating pathways through which self-efficacy is associated with work engagement. The study also underscores the relevance of PP in foreign language teaching, demonstrating that teachers’ emotional experiences, both positive and negative, are associated with work engagement, which has been linked to language teaching quality (MacIntyre, 2021) and teachers’ sustainable professional development (Cao, 2025; Høigaard et al., 2012).

The study holds several practical implications for stakeholders in language education. First, educational policymakers and school administrators are encouraged to consider implementing affective support systems, such as recognition schemes and structured peer-sharing opportunities, that may foster a positive school climate (Zhang et al., 2023a). These initiatives may help support teacher engagement by providing psychological and social resources and promoting teaching enjoyment. Second, teacher educators could consider designing targeted workshops and training sessions (Zhang et al., 2023a) to guide teachers in consciously recognizing, documenting, and reflecting on enjoyable teaching moments. Such training, when combined with strategies for managing teaching anxiety, may be particularly valuable for supporting engagement, as both emotional pathways appear to be equally important. In addition, school-level EFL teachers might find it helpful to practice emotional reflection practices such as keeping a journal to record both anxiety-provoking and enjoyment-related experiences. Such practices may help teachers reflect on their emotional experiences and support their professional engagement over time. Further experimental or intervention studies are needed to establish their effectiveness.

Three limitations should be acknowledged. First, the use of convenience and snowball sampling limits the representativeness of the sample, as seen in the over-representation of groups such as female and early-career teachers. Demographic variables were not included as covariates because they were not specified a priori in the theoretically driven model and because of the sample size and model complexity. Future research with more diverse samples and multigroup analyses is needed to examine demographic variations. Second, the reliance on self-report measures may introduce the risk of common method bias. Although Harman’s single-factor test showed that the first unrotated factor accounted for 40.39% of the variance, below the conventional 50% threshold, this test alone is insufficient to rule out common method bias. Future mixed-methods designs incorporating observational or interview data could provide additional sources of evidence. Third, the cross-sectional design precludes causal inferences. The proposed mediation model is theory-driven and statistically plausible, but alternative directional models (e.g., sequential mediation; reciprocal relationships) cannot be ruled out. Longitudinal or experimental designs are needed to test causal ordering. Collectively, these limitations suggest that the present findings, while informative, should be interpreted with appropriate caution. Future studies employing more representative sampling, mixed-methods designs, and longitudinal or experimental approaches are needed to extend and corroborate the current results.

## Figures and Tables

**Table 1:** Demographic information of the participants.

Categories	Sub-Categories		*n*	Percentage (%)
Gender	Male		66	22.92%
	Female		222	77.08%
Location of the Workplace	Urban Schools		199	69.1%
	Rural Schools		89	30.9%
Types of Working Schools	Primary School		101	35.07%
	Junior High School		107	37.15%
	Senior High School	Core	50	17.36%
		Ordinary	30	10.42%
Educational Qualifications	Bachelor’s degree		170	58.03%
	Master’s degree		95	32.99%
	Ph.D./Ed.D.		23	7.99%
Teaching Experience	1–3 years		115	39.93%
	4–6 years		56	19.44%
	7–18 years		85	29.51%
	19–30 years		27	9.38%
	More than 30 years		5	1.74%

**Table 2:** Descriptive Statistics, Correlations, and Discriminant Validity.

	M(SD)	Skewness	Kurtosis	1	2	3	4
TSE	3.59(0.84)	0.95	0.10	**0.766**			
FLTA	2.51(0.90)	0.61	0.49	−0.507 **	**0.728**		
FLTE	3.69(0.78)	−1.13	0.84	0.640 ***	−0.489 ***	**0.731**	
TWE	4.51(0.34)	0.74	0.40	0.639 ***	−0.657 ***	0.673 ***	**0.779**

Note: TSE = teacher self-efficacy; FLTA = foreign language teaching anxiety; FLTE = foreign language teaching enjoyment; TWE = teacher work engagement. Diagonal values (In bold) are the square roots of the AVE for each construct. Off-diagonal values are Pearson correlations among constructs. *** *p* < 0.001, ** *p* < 0.01.

**Table 3:** Measurement models for the four latent variables.

	χ^2^	df	χ^2^/df	CFI	TLI	RMSEA	AVE	CR
	-	-	<3.000	>0.900	>0.900	<0.080	>0.500	>0.700
TSE	141.215	54	2.615	0.963	0.954	0.075	0.586	0.944
FLTA	150.022	54	2.778	0.951	0.940	0.079	0.530	0.931
FLTE	60.019	27	2.223	0.975	0.967	0.065	0.534	0.911
TWE	58.183	27	2.155	0.981	0.975	0.063	0.607	0.933

Note. TSE = teacher self-efficacy; FLTA = foreign language teaching anxiety; FLTE = foreign language teaching enjoyment; TWE = teacher work engagement. AVE = average variance extracted; CR = composite reliability.

**Table 4:** Comparison of Measurement Models.

Model	χ^2^	df	χ^2^/df	CFI	TLI	RMSEA
	-	-	<3.000	>0.900	>0.900	<0.080
One-factor	3640.514	819	4.445	0.638	0.620	0.110
Two-factor	2967.244	818	3.627	0.725	0.710	0.096
Three-factor	2087.738	816	2.559	0.837	0.828	0.074
Four-factor (proposed)	1146.680	813	1.410	0.957	0.955	0.038

**Table 5:** The direct effects of SEM analysis.

Path	*β*	S.E.	C.R.	*p*	BC 95% CI (β)
TSE-TWE	0.232	0.108	3.759	0.023	[0.029, 0.452]
TSE-FLTA	−0.507	0.062	−7.212	<0.001	[−0.622, −0.375]
TSE-FLTE	0.640	0.067	8.357	<0.001	[0.498, 0.763]
FLTA-TWE	−0.373	0.074	−6.447	<0.001	[−0.515, −0.228]
FLTE-TWE	0.342	0.107	5.254	0.001	[0.142, 0.561]

Note. TSE = teacher self-efficacy; FLTA = foreign language teaching anxiety; FLTE = foreign language teaching enjoyment; TWE = teacher work engagement; *β* = Standardized path coefficient; S.E. = standard error (bootstrap standard error for standardized estimates); C.R. = critical ratio; BC 95% CI = bias-corrected bootstrap 95% confidence interval based on 5000 bootstrap samples.

**Table 6:** The results of the mediation analysis.

Path	*β*	*B*	BC 95% CI (*B*)	*p*
Indirect effects				
TSE-FLTA-TWE	0.189	0.360	[0.218, 0.573]	<0.001
TSE-FLTE-TWE	0.219	0.418	[0.175, 0.779]	0.001
Total indirect effect	0.408	0.779	[0.489, 1.170]	<0.001
Direct effect				
TSE-TWE	0.232	0.443	[0.057, 0.861]	0.023
Total effect				
TSE-TWE	0.639	1.221	[0.957, 1.535]	<0.001

Note. TSE = teacher self-efficacy; FLTA = foreign language teaching anxiety; FLTE = foreign language teaching enjoyment; TWE = teacher work engagement; *β* = Standardized path coefficient; B = unstandardized path coefficient; BC 95% CI = bias-corrected bootstrap 95% confidence interval based on 5000 bootstrap samples.

## Data Availability

The Data supporting the findings of this study can be obtained by contacting the first author.

## Abbreviations

The following abbreviations are used in this manuscript:

TSE	Teacher Self-Efficacy
FLTA	Foreign Language Teaching Anxiety
FLTE	Foreign Language Teaching Enjoyment
TWE	Teacher Work Engagement

## Appendix A. Standardized Factor Loadings for All Items

**Construct**	**Item**	**Standardized Loading**	***p***
Teacher Self-Efficacy (TSE)	Q9_Row1	0.689	<0.001
	Q9_Row2	0.759	<0.001
	Q9_Row3	0.786	<0.001
	Q9_Row4	0.794	<0.001
	Q9_Row5	0.681	<0.001
	Q9_Row6	0.702	<0.001
	Q9_Row7	0.758	<0.001
	Q9_Row8	0.847	<0.001
	Q9_Row9	0.796	<0.001
	Q9_Row10	0.737	<0.001
	Q9_Row11	0.815	<0.001
	Q9_Row12	0.802	<0.001
Foreign Language Teaching Anxiety (FLTA)	Q10_Row1	0.708	<0.001
	Q10_Row2	0.774	<0.001
	Q10_Row3	0.694	<0.001
	Q10_Row4	0.795	<0.001
	Q10_Row5	0.738	<0.001
	Q10_Row6	0.672	<0.001
	Q10_Row7	0.749	<0.001
	Q10_Row8	0.771	<0.001
	Q10_Row9	0.618	<0.001
	Q10_Row10	0.784	<0.001
	Q10_Row11	0.665	<0.001
	Q10_Row12	0.747	<0.001
Foreign Language Teaching Enjoyment (FLTE)	Q11_Row1	0.679	<0.001
	Q11_Row2	0.775	<0.001
	Q11_Row3	0.743	<0.001
	Q11_Row4	0.775	<0.001
	Q11_Row5	0.643	<0.001
	Q11_Row6	0.662	<0.001
	Q11_Row7	0.724	<0.001
	Q11_Row8	0.769	<0.001
	Q11_Row9	0.788	<0.001
Teacher Work Engagement (TWE)	Q12_Row1	0.817	<0.001
	Q12_Row2	0.792	<0.001
	Q12_Row3	0.772	<0.001
	Q12_Row4	0.782	<0.001
	Q12_Row5	0.731	<0.001
	Q12_Row6	0.779	<0.001
	Q12_Row7	0.837	<0.001
	Q12_Row8	0.787	<0.001
	Q12_Row9	0.707	<0.001

## References

[B1-behavsci-16-01640] Bandura A. (1977). Self-efficacy: Toward a unifying theory of behavioral change. Psychological Review.

[B2-behavsci-16-01640] Bandura A. (1997). Self-efficacy: The exercise of control.

[B3-behavsci-16-01640] Cao G. (2025). Chinese EFL teachers’ professional agency as a facilitator of their sustainable development and work engagement. International Journal of Applied Linguistics.

[B4-behavsci-16-01640] Derakhshan A., Greenier V., Fathi J. (2023). Exploring the interplay between a loving pedagogy, creativity, and work engagement among EFL/ESL teachers: A multinational study. Current Psychology.

[B5-behavsci-16-01640] Dewaele J., MacIntyre P., Sato M., Loewen S. (2019). The predictive power of multicultural personality traits, learner and teacher variables on foreign language enjoyment and anxiety. Evidence-based second language pedagogy: A collection of instructed second language acquisition studies.

[B6-behavsci-16-01640] Dörnyei Z. (2007). Research methods in applied linguistics: Quantitative, qualitative, and mixed methodologies.

[B7-behavsci-16-01640] Fan J., Lu X., Zhang Q. (2024). The impact of teacher and peer support on preservice EFL teachers’ work engagement in their teaching practicum: The mediating role of teacher L2 grit and language teaching enjoyment. Behavioral Sciences.

[B8-behavsci-16-01640] Fan Y. G. (2022). Reviewing the effect of English as a foreign language teachers’ positive and negative affectivity on their work engagement. Frontiers in Psychology.

[B9-behavsci-16-01640] Fathi J., Zhang L. J., Arefian M. (2023). Testing a model of EFL teachers’ work engagement: The roles of teachers’ professional identity, L2 grit, and foreign language teaching enjoyment. Review of Applied Linguistics in Language Teaching.

[B10-behavsci-16-01640] Fraschini N., Park H. (2021). Anxiety in language teachers: Exploring the variety of perceptions with Q methodology. Foreign Language Annals.

[B11-behavsci-16-01640] Fredrickson B. L. (2001). The role of positive emotions in positive psychology: The broaden-and-build theory of positive emotions. American Psychologist.

[B12-behavsci-16-01640] Fredrickson B. L. (2003). The value of positive emotions: The emerging science of positive psychology is coming to understand why it’s good to feel good. American Scientist.

[B13-behavsci-16-01640] Fuller C. M., Simmering M. J., Atinc G., Atinc Y. O., Babin B. J. (2016). Common methods variance detection in business research. Journal of Business Research.

[B14-behavsci-16-01640] Geng X., Han J. (2025). Individual differences in interpersonal emotion regulation: What makes some foreign language teachers more or less successful in their relational well-being at work?. International Journal of Educational Research.

[B15-behavsci-16-01640] Gong K., Liu M., Hu W. (2025). Emotions in constructing teaching self-image: Mediation models of Chinese university EFL teachers’ anxiety and self-efficacy. International Journal of Applied Linguistics.

[B16-behavsci-16-01640] Gregersen T., Mercer S. (2021). The Routledge handbook of the psychology of language learning and teaching.

[B17-behavsci-16-01640] Hakanen J. J., Bakker A. B., Schaufeli W. B. (2006). Burnout and work engagement among teachers. Journal of School Psychology.

[B18-behavsci-16-01640] Hayes A. F. (2013). Introduction to mediation, moderation, and conditional process analysis: A regression-based approach.

[B19-behavsci-16-01640] Horwitz E. K. (1996). Even Teachers Get the Blues: Recognizing and Alleviating Language Teachers’ Feelings of Foreign Language Anxiety. Foreign Language Annals.

[B20-behavsci-16-01640] Høigaard R., Giske R., Sundsli K. (2012). Newly qualified teachers’ work engagement and teacher efficacy influences on job satisfaction, burnout, and the intention to quit. European Journal of Teacher Education.

[B21-behavsci-16-01640] Kim S., Kim J. (2004). When the learner becomes a teacher: Foreign language anxiety as an occupational hazard. English Teaching.

[B22-behavsci-16-01640] Klassen R. M., Aldhafri S., Mansfield C. F., Purwanto E., Siu A. F. Y., Wong M. W., Woods-McConney A. (2012). Teachers’ engagement at work: An international validation study. Journal of Experimental Education.

[B23-behavsci-16-01640] Kline R. B. (2016). Principles and practice of structural equation modeling.

[B24-behavsci-16-01640] Kunnan A. (1998). An introduction to structural equation modeling for language assessment research. Language Testing.

[B25-behavsci-16-01640] Liu H., Chen B., Li X., Zhou X. (2024). Exploring the predictive role of self-efficacy in engagement among EFL teachers in online teaching: The mediation of buoyancy. The Asia-Pacific Education Researcher.

[B26-behavsci-16-01640] Liu M., Wu B. (2021). Teaching anxiety and foreign language anxiety among Chinese college English teachers. SAGE Open.

[B27-behavsci-16-01640] MacIntyre P. D., Budzińska K., Majchrzak O. (2021). Exploring applications of positive psychology in SLA. Positive psychology in second and foreign language education.

[B28-behavsci-16-01640] Mișu S. I., Radu C., Deaconu A., Toma S. (2022). How to increase teacher performance through engagement and work efficacy. Sustainability.

[B29-behavsci-16-01640] Oxford R. L., MacIntyre P. D., Gregersen T., Mercer S. (2016). Toward a psychology of well-being for language learners: The ‘EMPATHICS’ vision. Positive psychology in SLA.

[B30-behavsci-16-01640] Podsakoff P. M., MacKenzie S. B., Lee J. Y., Podsakoff N. P. (2003). Common method biases in behavioral research: A critical review of the literature and recommended remedies. Journal of Applied Psychology.

[B31-behavsci-16-01640] Proietti Ergün A. L., Dewaele J. (2021). Do well-being and resilience predict the foreign language teaching enjoyment of teachers of Italian?. System.

[B32-behavsci-16-01640] Räsänen K., Pietarinen J., Pyhältö K., Soini T., Väisänen P. (2020). Why leave the teaching profession? A longitudinal approach to the prevalence and persistence of teacher turnover intentions. Social Psychology of Education.

[B33-behavsci-16-01640] Schaufeli W. B., Bakker A. B., Salanova M. (2006). The measurement of work engagement with a short questionnaire: A cross-national study. Educational and Psychological Measurement.

[B34-behavsci-16-01640] Schreiber J. B., Stage F. K., King J., Nora A., Barlow E. A. (2006). Reporting structural equation modeling and confirmatory factor analysis results: A review. The Journal of Educational Research.

[B35-behavsci-16-01640] Seligman M. E. P., Csikszentmihalyi M. (2000). Positive psychology: An introduction. American Psychologist.

[B36-behavsci-16-01640] Tabachnick B. G., Fidell L. S. (2013). Using multivariate statistics.

[B37-behavsci-16-01640] Tschannen-Moran M., Hoy A. W. (2001). Teacher efficacy: Capturing an elusive construct. Teaching and Teacher Education.

[B38-behavsci-16-01640] Wang Y., Derakhshan A., Noughabi M. A. (2022). The interplay of EFL teachers’ immunity, work engagement, and psychological well-being: Evidence from four Asian countries. Journal of Multilingual and Multicultural Development.

[B39-behavsci-16-01640] Wang Y., Derakhshan A., Solhi M. (2025). Dispositions toward loving pedagogy, emotion regulation, and self-efficacy as predictors of EFL teachers’ work engagement: A multinational study. Language Teaching Research.

[B40-behavsci-16-01640] Wu S.-M. (2025). The relationships of workplace spirituality and psychological capital with work engagement among junior high school teachers. Behavioral Sciences.

[B41-behavsci-16-01640] Wu Y., Zhao D., Zhao Q. (2026). Does smashing teachers’ iron rice bowl stimulate their work engagement? The mediating role of Chinese traditional qunji values in teacher personnel reform. International Journal of Educational Research.

[B42-behavsci-16-01640] Xiao Y., Fathi J., Mohammaddokht F. (2022). Exploring a structural model of teaching enjoyment, teacher self-efficacy, and work engagement. Frontiers in Psychology.

[B43-behavsci-16-01640] Xu R., Jia X. (2022). An investigation into Chinese EFL teachers’ self-efficacy and stress as predictors of engagement and emotional exhaustion. SAGE Open.

[B44-behavsci-16-01640] Yang L., Duan M. (2024). Exploring the predictive role of job satisfaction on bilingual English teachers’ aggression and stress in the Chinese EFL context: A latent growth curve modelling. Journal of Multilingual and Multicultural Development.

[B45-behavsci-16-01640] Yang S., Noughabi M. A., Botes E., Dewaele J.-M. (2023). Let’s get positive: How foreign language teaching enjoyment can create a positive feedback loop. Studies in Second Language Learning and Teaching.

[B46-behavsci-16-01640] Zhang L. J., Fathi J., Mohammaddokht F. (2023a). Predicting teaching enjoyment from teachers’ perceived school climate, self-efficacy, and psychological wellbeing at work: EFL teachers. Perceptual and Motor Skills.

[B47-behavsci-16-01640] Zhang L. J., Fathi J., Naderi M. (2023b). A cross-lagged panel analysis of self-efficacy, teacher grit, teaching enjoyment, and work engagement among foreign language teachers. Journal of Multilingual and Multicultural Development.

[B48-behavsci-16-01640] Zhi R., Wang Y., Derakhshan A. (2024). On the role of academic buoyancy and self-efficacy in predicting teachers’ work engagement: A case of Chinese English as a foreign language teachers. Perceptual and Motor Skills.

